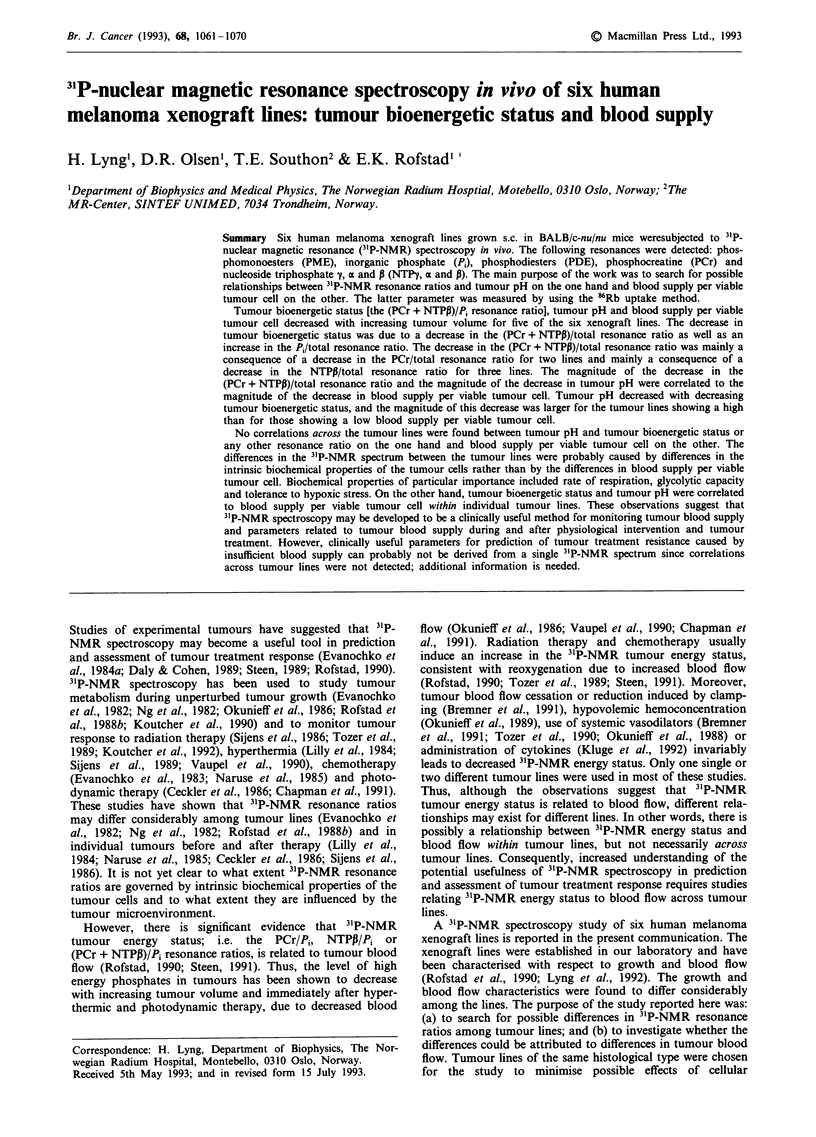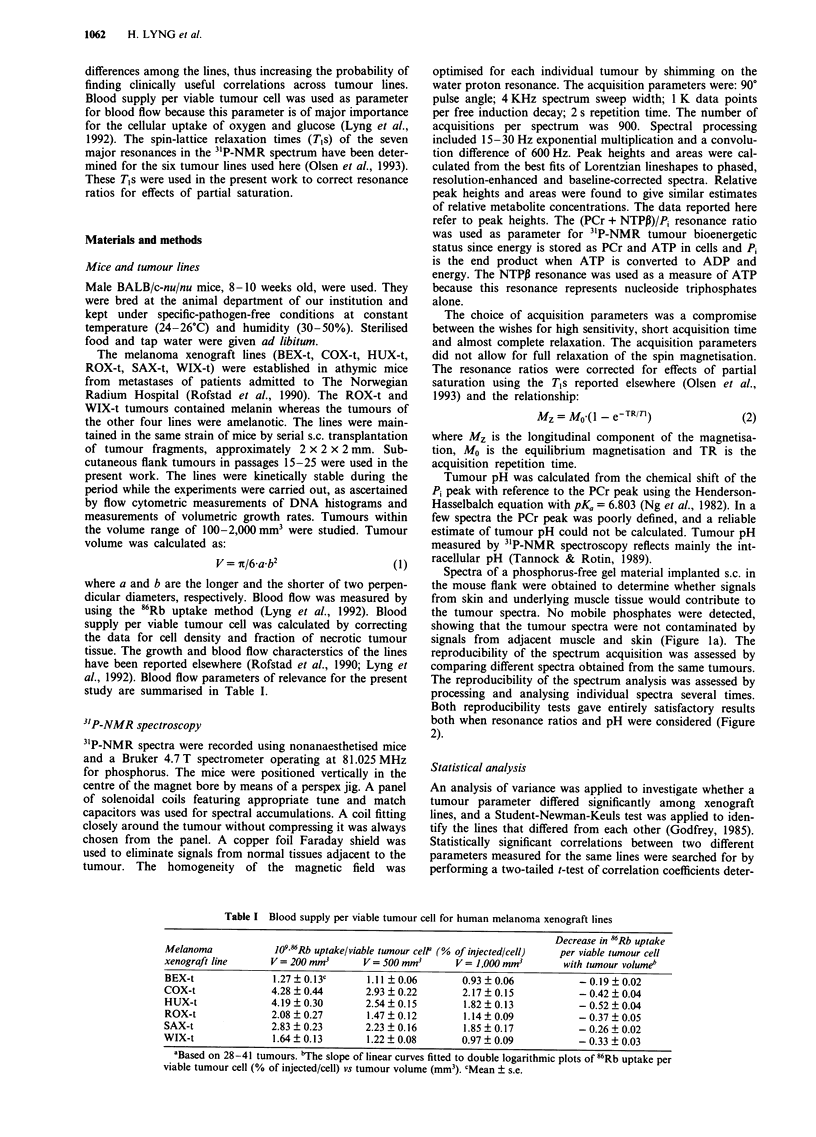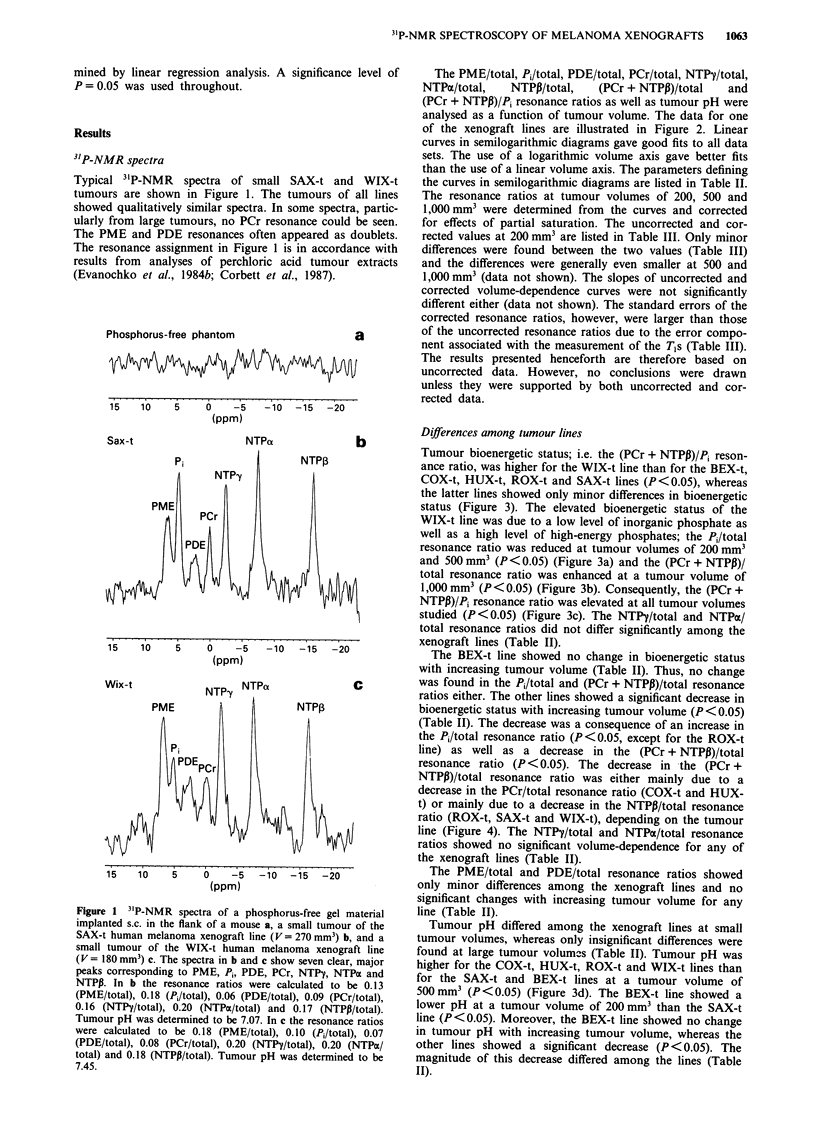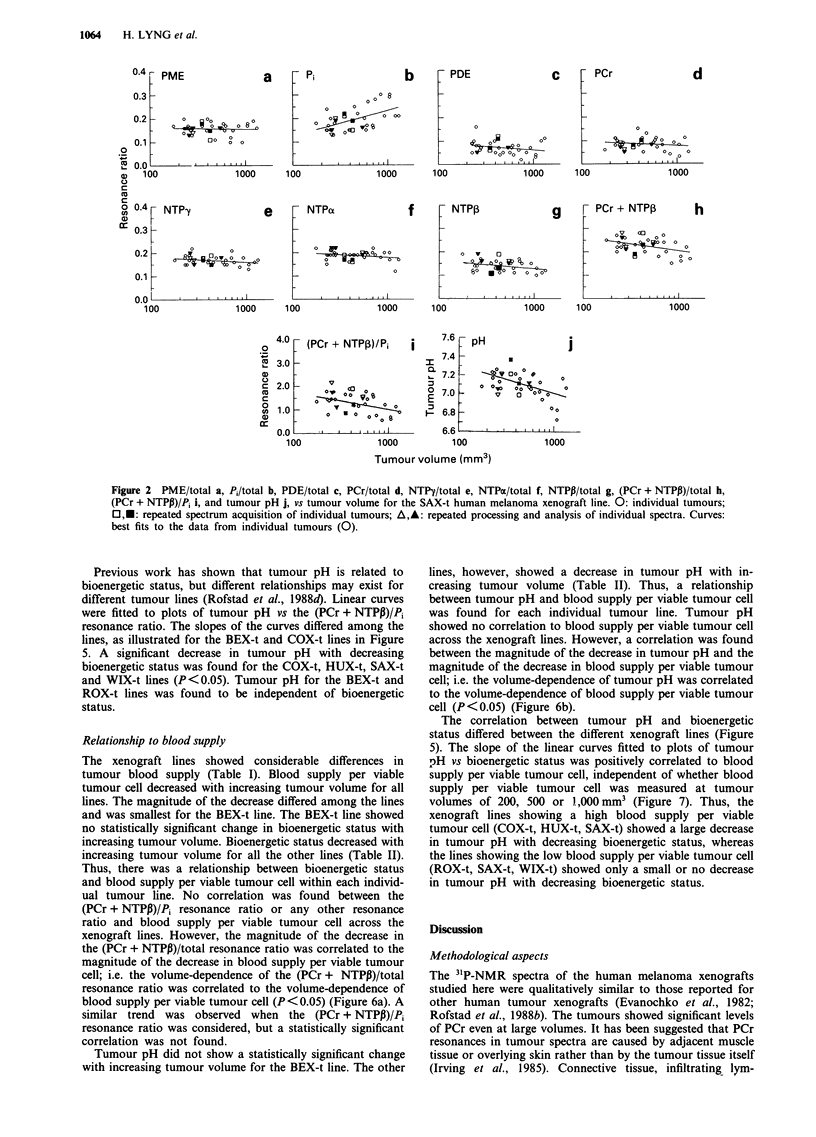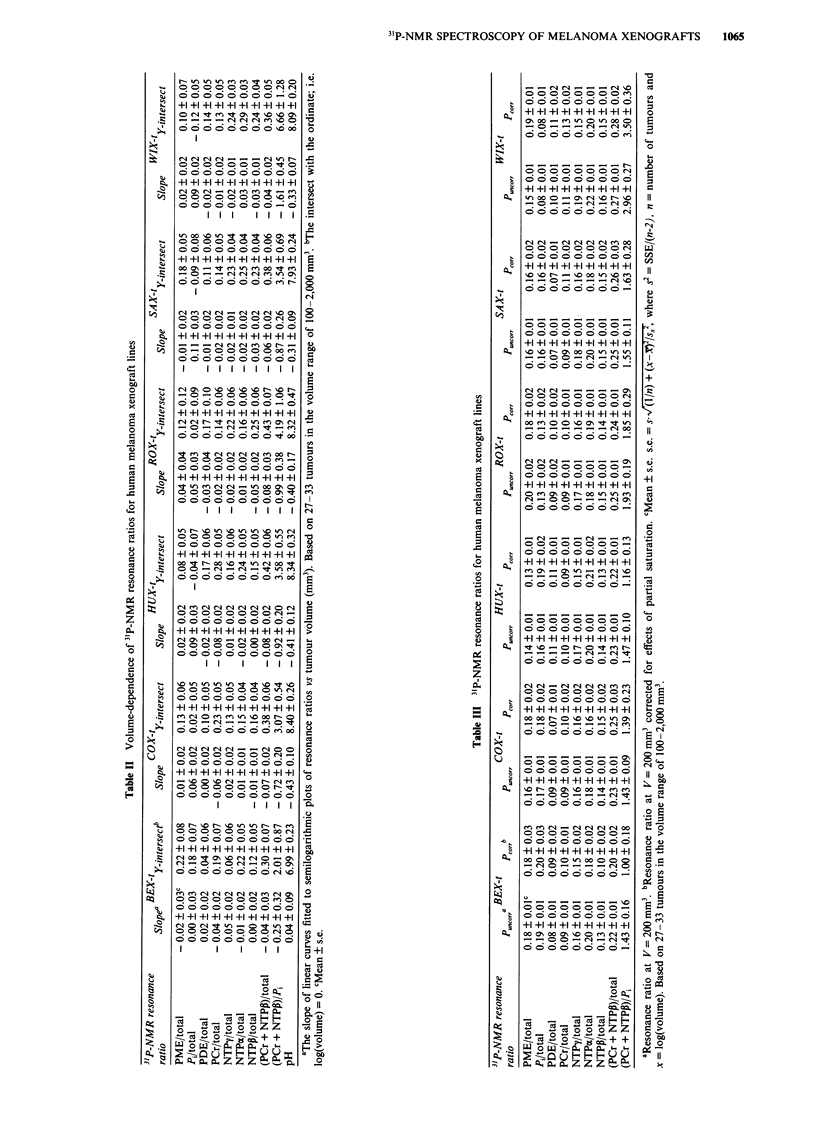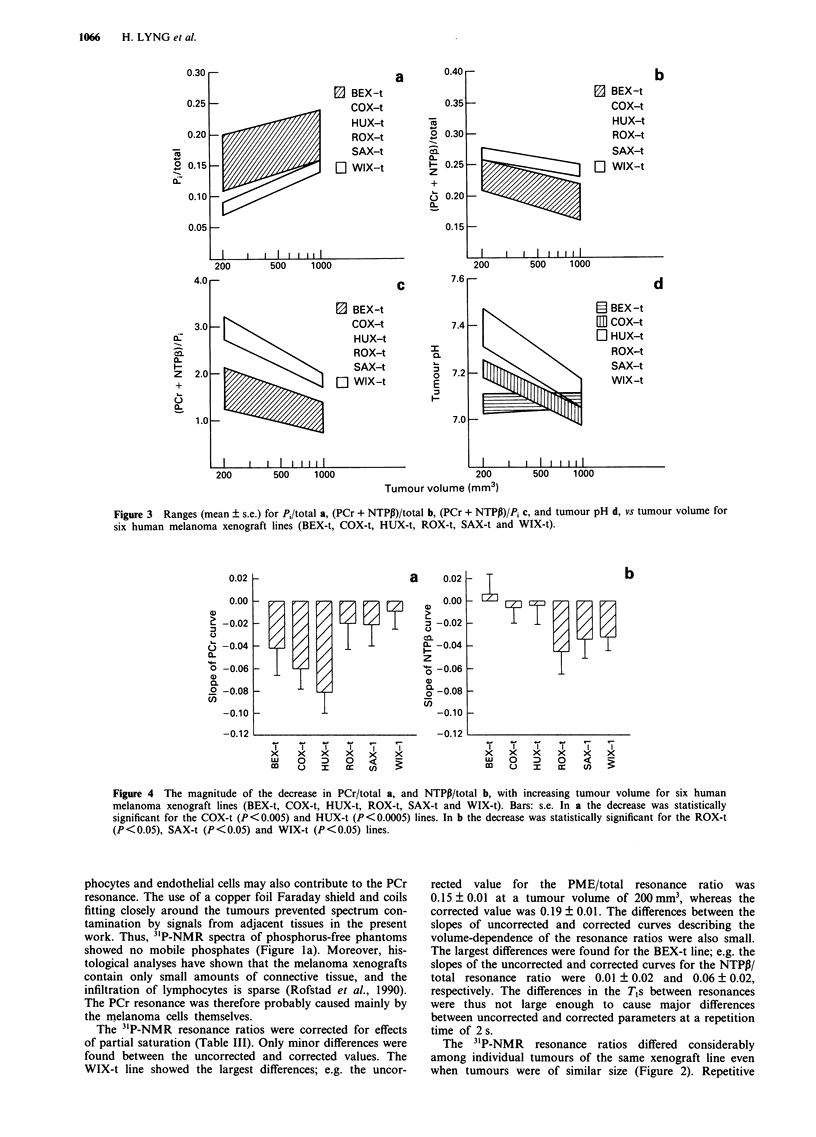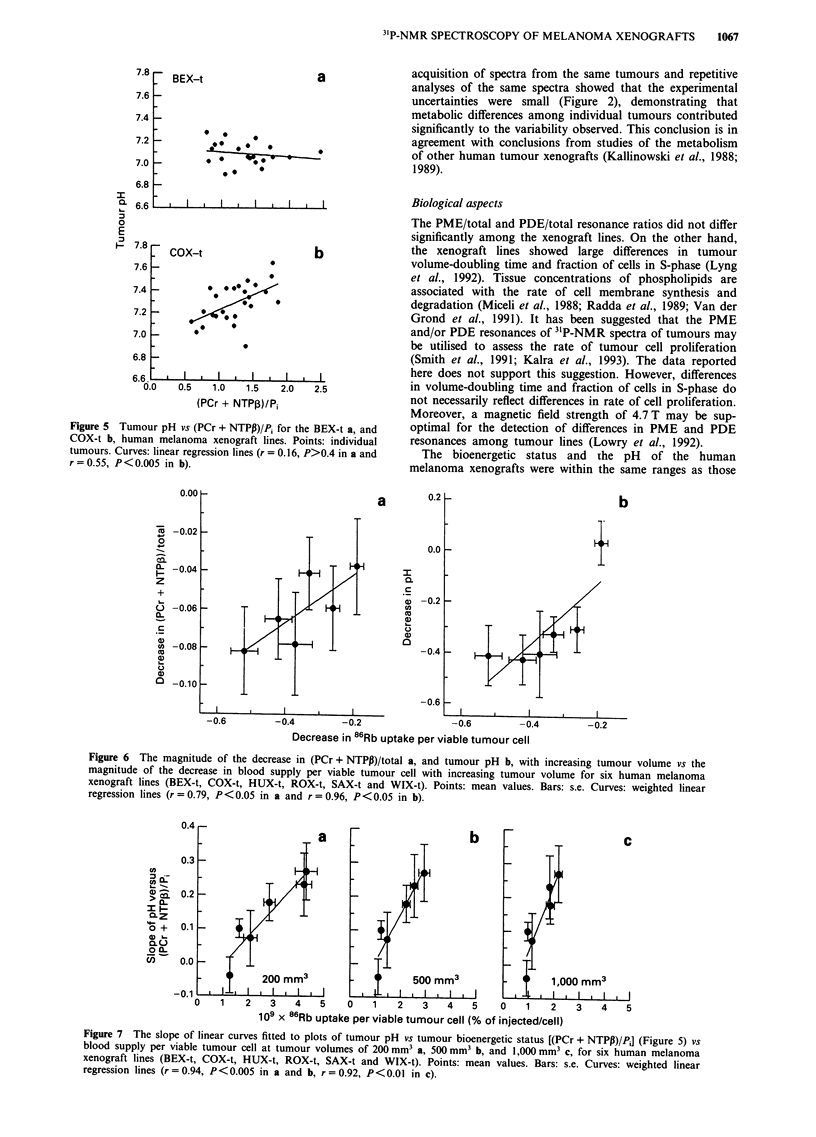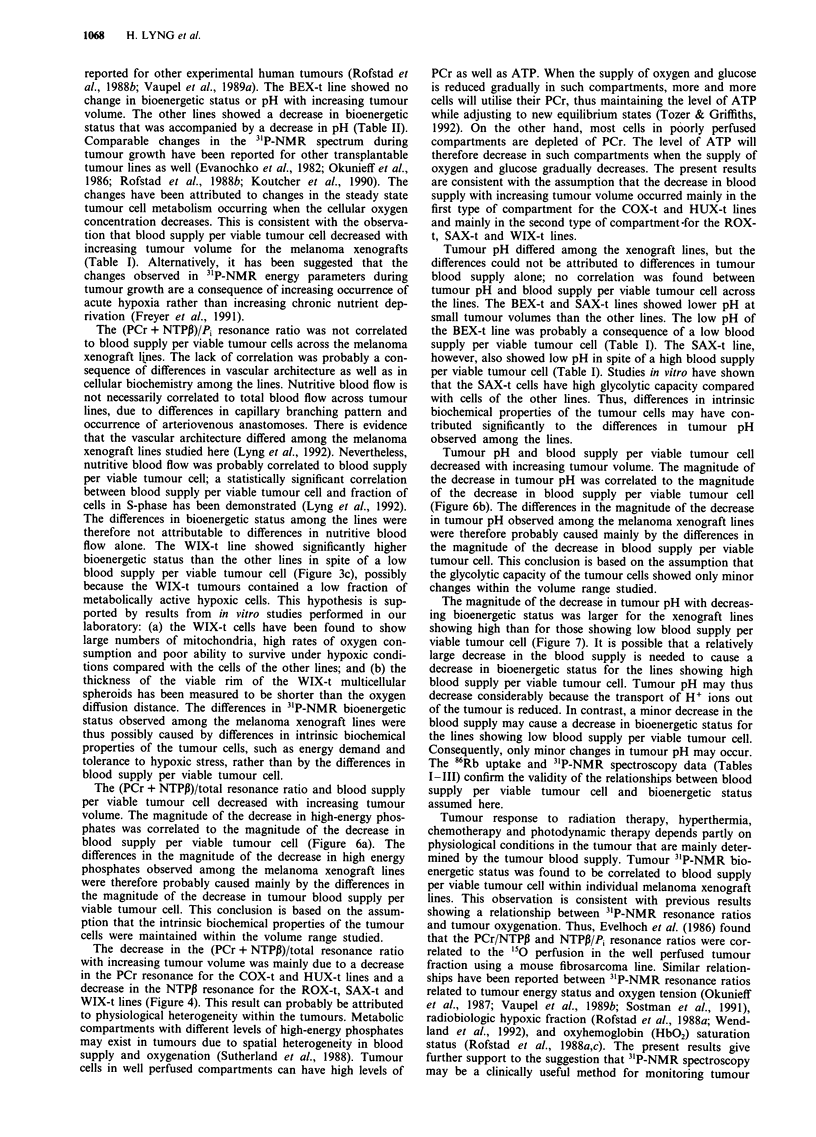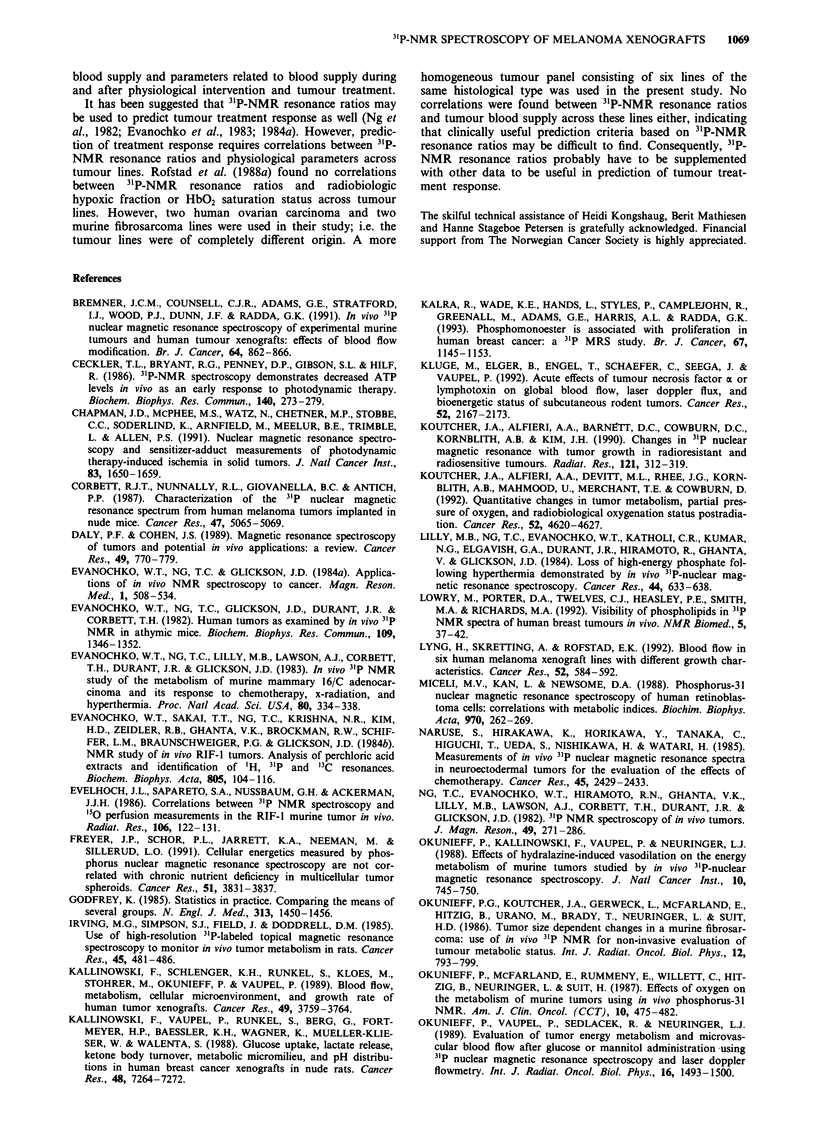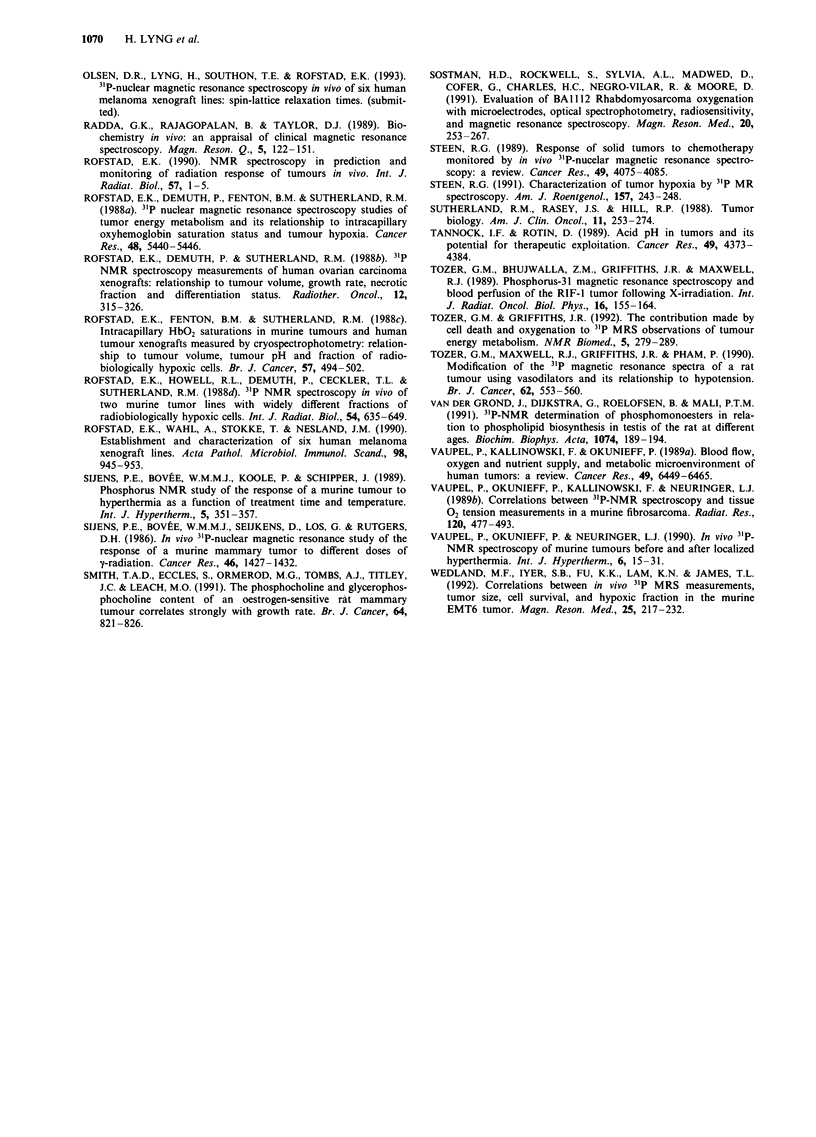# 31P-nuclear magnetic resonance spectroscopy in vivo of six human melanoma xenograft lines: tumour bioenergetic status and blood supply.

**DOI:** 10.1038/bjc.1993.483

**Published:** 1993-12

**Authors:** H. Lyng, D. R. Olsen, T. E. Southon, E. K. Rofstad

**Affiliations:** Department of Biophysics and Medical Physics, Norwegian Radium Hospital, Motebello, Oslo.

## Abstract

Six human melanoma xenograft lines grown s.c. in BALB/c-nu/nu mice were subjected to 31P-nuclear magnetic resonance (31P-NMR) spectroscopy in vivo. The following resonances were detected: phosphomonoesters (PME), inorganic phosphate (Pi), phosphodiesters (PDE), phosphocreatine (PCr) and nucleoside triphosphate gamma, alpha and beta (NTP gamma, alpha and beta). The main purpose of the work was to search for possible relationships between 31P-NMR resonance ratios and tumour pH on the one hand and blood supply per viable tumour cell on the other. The latter parameter was measured by using the 86Rb uptake method. Tumour bioenergetic status [the (PCr + NTP beta)/Pi resonance ratio], tumour pH and blood supply per viable tumour cell decreased with increasing tumour volume for five of the six xenograft lines. The decrease in tumour bioenergetic status was due to a decrease in the (PCr + NTP beta)/total resonance ratio as well as an increase in the Pi/total resonance ratio. The decrease in the (PCr + NTP beta)/total resonance ratio was mainly a consequence of a decrease in the PCr/total resonance ratio for two lines and mainly a consequence of a decrease in the NTP beta/total resonance ratio for three lines. The magnitude of the decrease in the (PCr + NTP beta)/total resonance ratio and the magnitude of the decrease in tumour pH were correlated to the magnitude of the decrease in blood supply per viable tumour cell. Tumour pH decreased with decreasing tumour bioenergetic status, and the magnitude of this decrease was larger for the tumour lines showing a high than for those showing a low blood supply per viable tumour cell. No correlations across the tumour lines were found between tumour pH and tumour bioenergetic status or any other resonance ratio on the one hand and blood supply per viable tumour cell on the other. The differences in the 31P-NMR spectrum between the tumour lines were probably caused by differences in the intrinsic biochemical properties of the tumour cells rather than by the differences in blood supply per viable tumour cell. Biochemical properties of particular importance included rate of respiration, glycolytic capacity and tolerance to hypoxic stress. On the other hand, tumour bioenergetic status and tumour pH were correlated to blood supply per viable tumour cell within individual tumour lines. These observations suggest that 31P-NMR spectroscopy may be developed to be a clinically useful method for monitoring tumour blood supply and parameters related to tumour blood supply during and after physiological intervention and tumour treatment. However, clinically useful parameters for prediction of tumour treatment resistance caused by insufficient blood supply can probably not be derived from a single 31P-NMR spectrum since correlations across tumour lines were not detected; additional information is needed.


Br. J. Cancer (1993), 68, 1061–1070 © Macmillan Press Ltd., 1993

### [31]P-nuclear magnetic resonance spectroscopy *in vivo* of six human melanoma xenograft lines: tumour bioenergetic status and blood supply

H. Lyng[1], D.R. Olsen[1], T.E. Southon[2] & E.K. Rofstad[1]

[1]*Department of Biophysics and Medical Physics, The Norwegian Radium Hosptial, Motebello, 0310 Oslo, Norway;* [2]*The MR-Center, SINTEF UNIMED, 7034 Trondheim, Norway.*

**Summary** Six human melanoma xenograft lines grown s.c. in BALB/c-*nu/nu* mice weresubjected to [31]P-nuclear magnetic resonance ([31]P-NMR) spectroscopy *in vivo*. The following resonances were detected: phosphomonoesters (PME), inorganic phosphate ($P_i$), phosphodiesters (PDE), phosphocreatine (PCr) and nucleoside triphosphate $\gamma$, $\alpha$ and $\beta$ (NTP$\gamma$, $\alpha$ and $\beta$). The main purpose of the work was to search for possible relationships between [31]P-NMR resonance ratios and tumour pH on the one hand and blood supply per viable tumour cell on the other. The latter parameter was measured by using the [86]Rb uptake method.

Tumour bioenergetic status [the (PCr + NTP$\beta$)/$P_i$ resonance ratio], tumour pH and blood supply per viable tumour cell decreased with increasing tumour volume for five of the six xenograft lines. The decrease in tumour bioenergetic status was due to a decrease in the (PCr + NTP$\beta$)/total resonance ratio as well as an increase in the $P_i$/total resonance ratio. The decrease in the (PCr + NTP$\beta$)/total resonance ratio was mainly a consequence of a decrease in the PCr/total resonance ratio for two lines and mainly a consequence of a decrease in the NTP$\beta$/total resonance ratio for three lines. The magnitude of the decrease in the (PCr + NTP$\beta$)/total resonance ratio and the magnitude of the decrease in tumour pH were correlated to the magnitude of the decrease in blood supply per viable tumour cell. Tumour pH decreased with decreasing tumour bioenergetic status, and the magnitude of this decrease was larger for the tumour lines showing a high than for those showing a low blood supply per viable tumour cell.

No correlations *across* the tumour lines were found between tumour pH and tumour bioenergetic status or any other resonance ratio on the one hand and blood supply per viable tumour cell on the other. The differences in the [31]P-NMR spectrum between the tumour lines were probably caused by differences in the intrinsic biochemical properties of the tumour cells rather than by the differences in blood supply per viable tumour cell. Biochemical properties of particular importance included rate of respiration, glycolytic capacity and tolerance to hypoxic stress. On the other hand, tumour bioenergetic status and tumour pH were correlated to blood supply per viable tumour cell *within* individual tumour lines. These observations suggest that [31]P-NMR spectroscopy may be developed to be a clinically useful method for monitoring tumour blood supply and parameters related to tumour blood supply during and after physiological intervention and tumour treatment. However, clinically useful parameters for prediction of tumour treatment resistance caused by insufficient blood supply can probably not be derived from a single [31]P-NMR spectrum since correlations across tumour lines were not detected; additional information is needed.

Studies of experimental tumours have suggested that [31]P-NMR spectroscopy may become a useful tool in prediction and assessment of tumour treatment response (Evanochko *et al.*, 1984*a*; Daly & Cohen, 1989; Steen, 1989; Rofstad, 1990). [31]P-NMR spectroscopy has been used to study tumour metabolism during unperturbed tumour growth (Evanochko *et al.*, 1982; Ng *et al.*, 1982; Okunieff *et al.*, 1986; Rofstad *et al.*, 1988*b*; Koutcher *et al.*, 1990) and to monitor tumour response to radiation therapy (Sijens *et al.*, 1986; Tozer *et al.*, 1989; Koutcher *et al.*, 1992), hyperthermia (Lilly *et al.*, 1984; Sijens *et al.*, 1989; Vaupel *et al.*, 1990), chemotherapy (Evanochko *et al.*, 1983; Naruse *et al.*, 1985) and photodynamic therapy (Ceckler *et al.*, 1986; Chapman *et al.*, 1991). These studies have shown that [31]P-NMR resonance ratios may differ considerably among tumour lines (Evanochko *et al.*, 1982; Ng *et al.*, 1982; Rofstad *et al.*, 1988*b*) and in individual tumours before and after therapy (Lilly *et al.*, 1984; Naruse *et al.*, 1985; Ceckler *et al.*, 1986; Sijens *et al.*, 1986). It is not yet clear to what extent [31]P-NMR resonance ratios are governed by intrinsic biochemical properties of the tumour cells and to what extent they are influenced by the tumour microenvironment.

However, there is significant evidence that [31]P-NMR tumour energy status; i.e. the PCr/$P_i$, NTP$\beta$/$P_i$ or (PCr + NTP$\beta$)/$P_i$ resonance ratios, is related to tumour blood flow (Rofstad, 1990; Steen, 1991). Thus, the level of high energy phosphates in tumours has been shown to decrease with increasing tumour volume and immediately after hyperthermic and photodynamic therapy, due to decreased blood flow (Okunieff *et al.*, 1986; Vaupel *et al.*, 1990; Chapman *et al.*, 1991). Radiation therapy and chemotherapy usually induce an increase in the [31]P-NMR tumour energy status, consistent with reoxygenation due to increased blood flow (Rofstad, 1990; Tozer *et al.*, 1989; Steen, 1991). Moreover, tumour blood flow cessation or reduction induced by clamping (Bremner *et al.*, 1991), hypovolemic hemoconcentration (Okunieff *et al.*, 1989), use of systemic vasodilators (Bremner *et al.*, 1991; Tozer *et al.*, 1990; Okunieff *et al.*, 1988) or administration of cytokines (Kluge *et al.*, 1992) invariably leads to decreased [31]P-NMR energy status. Only one single or two different tumour lines were used in most of these studies. Thus, although the observations suggest that [31]P-NMR tumour energy status is related to blood flow, different relationships may exist for different lines. In other words, there is possibly a relationship between [31]P-NMR energy status and blood flow *within* tumour lines, but not necessarily *across* tumour lines. Consequently, increased understanding of the potential usefulness of [31]P-NMR spectroscopy in prediction and assessment of tumour treatment response requires studies relating [31]P-NMR energy status to blood flow across tumour lines.

A [31]P-NMR spectroscopy study of six human melanoma xenograft lines is reported in the present communication. The xenograft lines were established in our laboratory and have been characterised with respect to growth and blood flow (Rofstad *et al.*, 1990; Lyng *et al.*, 1992). The growth and blood flow characteristics were found to differ considerably among the lines. The purpose of the study reported here was: (a) to search for possible differences in [31]P-NMR resonance ratios among tumour lines; and (b) to investigate whether the differences could be attributed to differences in tumour blood flow. Tumour lines of the same histological type were chosen for the study to minimise possible effects of cellular

Correspondence: H. Lyng, Department of Biophysics, The Norwegian Radium Hospital, Montebello, 0310 Oslo, Norway.
Received 5th May 1993; and in revised form 15 July 1993.

differences among the lines, thus increasing the probability of finding clinically useful correlations across tumour lines. Blood supply per viable tumour cell was used as parameter for blood flow because this parameter is of major importance for the cellular uptake of oxygen and glucose (Lyng et al., 1992). The spin-lattice relaxation times ($T_1$s) of the seven major resonances in the $^{31}$P-NMR spectrum have been determined for the six tumour lines used here (Olsen et al., 1993). These $T_1$s were used in the present work to correct resonance ratios for effects of partial saturation.

### Materials and methods

### Mice and tumour lines

Male BALB/c-*nu/nu* mice, 8–10 weeks old, were used. They were bred at the animal department of our institution and kept under specific-pathogen-free conditions at constant temperature (24–26°C) and humidity (30–50%). Sterilised food and tap water were given *ad libitum*.

The melanoma xenograft lines (BEX-t, COX-t, HUX-t, ROX-t, SAX-t, WIX-t) were established in athymic mice from metastases of patients admitted to The Norwegian Radium Hospital (Rofstad et al., 1990). The ROX-t and WIX-t tumours contained melanin whereas the tumours of the other four lines were amelanotic. The lines were maintained in the same strain of mice by serial s.c. transplantation of tumour fragments, approximately 2 × 2 × 2 mm. Subcutaneous flank tumours in passages 15–25 were used in the present work. The lines were kinetically stable during the period while the experiments were carried out, as ascertained by flow cytometric measurements of DNA histograms and measurements of volumetric growth rates. Tumours within the volume range of 100–2,000 mm$^3$ were studied. Tumour volume was calculated as:

$$V = \pi/6 \cdot a \cdot b^2 \qquad (1)$$

where $a$ and $b$ are the longer and the shorter of two perpendicular diameters, respectively. Blood flow was measured by using the $^{86}$Rb uptake method (Lyng et al., 1992). Blood supply per viable tumour cell was calculated by correcting the data for cell density and fraction of necrotic tumour tissue. The growth and blood flow characterstics of the lines have been reported elsewhere (Rofstad et al., 1990; Lyng et al., 1992). Blood flow parameters of relevance for the present study are summarised in Table I.

### $^{31}$P-NMR spectroscopy

$^{31}$P-NMR spectra were recorded using nonanaesthetised mice and a Bruker 4.7 T spectrometer operating at 81.025 MHz for phosphorus. The mice were positioned vertically in the centre of the magnet bore by means of a perspex jig. A panel of solenoidal coils featuring appropriate tune and match capacitors was used for spectral accumulations. A coil fitting closely around the tumour without compressing it was always chosen from the panel. A copper foil Faraday shield was used to eliminate signals from normal tissues adjacent to the tumour. The homogeneity of the magnetic field was optimised for each individual tumour by shimming on the water proton resonance. The acquisition parameters were: 90° pulse angle; 4 KHz spectrum sweep width; 1 K data points per free induction decay; 2 s repetition time. The number of acquisitions per spectrum was 900. Spectral processing included 15–30 Hz exponential multiplication and a convolution difference of 600 Hz. Peak heights and areas were calculated from the best fits of Lorentzian lineshapes to phased, resolution-enhanced and baseline-corrected spectra. Relative peak heights and areas were found to give similar estimates of relative metabolite concentrations. The data reported here refer to peak heights. The (PCr + NTPβ)/$P_i$ resonance ratio was used as parameter for $^{31}$P-NMR tumour bioenergetic status since energy is stored as PCr and ATP in cells and $P_i$ is the end product when ATP is converted to ADP and energy. The NTPβ resonance was used as a measure of ATP because this resonance represents nucleoside triphosphates alone.

The choice of acquisition parameters was a compromise between the wishes for high sensitivity, short acquisition time and almost complete relaxation. The acquisition parameters did not allow for full relaxation of the spin magnetisation. The resonance ratios were corrected for effects of partial saturation using the $T_1$s reported elsewhere (Olsen et al., 1993) and the relationship:

$$M_Z = M_0 \cdot (1 - e^{-TR/T1}) \qquad (2)$$

where $M_Z$ is the longitudinal component of the magnetisation, $M_0$ is the equilibrium magnetisation and TR is the acquisition repetition time.

Tumour pH was calculated from the chemical shift of the $P_i$ peak with reference to the PCr peak using the Henderson-Hasselbalch equation with $pK_a = 6.803$ (Ng et al., 1982). In a few spectra the PCr peak was poorly defined, and a reliable estimate of tumour pH could not be calculated. Tumour pH measured by $^{31}$P-NMR spectroscopy reflects mainly the intracellular pH (Tannock & Rotin, 1989).

Spectra of a phosphorus-free gel material implanted s.c. in the mouse flank were obtained to determine whether signals from skin and underlying muscle tissue would contribute to the tumour spectra. No mobile phosphates were detected, showing that the tumour spectra were not contaminated by signals from adjacent muscle and skin (Figure 1a). The reproducibility of the spectrum acquisition was assessed by comparing different spectra obtained from the same tumours. The reproducibility of the spectrum analysis was assessed by processing and analysing individual spectra several times. Both reproducibility tests gave entirely satisfactory results both when resonance ratios and pH were considered (Figure 2).

### Statistical analysis

An analysis of variance was applied to investigate whether a tumour parameter differed significantly among xenograft lines, and a Student-Newman-Keuls test was applied to identify the lines that differed from each other (Godfrey, 1985). Statistically significant correlations between two different parameters measured for the same lines were searched for by performing a two-tailed $t$-test of correlation coefficients deter-

**Table I** Blood supply per viable tumour cell for human melanoma xenograft lines

| Melanoma xenograft line | $10^9 \cdot {}^{86}$Rb uptake/viable tumour cell[a] (% of injected/cell) V = 200 mm$^3$ | V = 500 mm$^3$ | V = 1,000 mm$^3$ | Decrease in $^{86}$Rb uptake per viable tumour cell with tumour volume[b] |
|---|---|---|---|---|
| BEX-t | 1.27 ± 0.13[c] | 1.11 ± 0.06 | 0.93 ± 0.06 | − 0.19 ± 0.02 |
| COX-t | 4.28 ± 0.44 | 2.93 ± 0.22 | 2.17 ± 0.15 | − 0.42 ± 0.04 |
| HUX-t | 4.19 ± 0.30 | 2.54 ± 0.15 | 1.82 ± 0.13 | − 0.52 ± 0.04 |
| ROX-t | 2.08 ± 0.27 | 1.47 ± 0.12 | 1.14 ± 0.09 | − 0.37 ± 0.05 |
| SAX-t | 2.83 ± 0.23 | 2.23 ± 0.16 | 1.85 ± 0.17 | − 0.26 ± 0.02 |
| WIX-t | 1.64 ± 0.13 | 1.22 ± 0.08 | 0.97 ± 0.09 | − 0.33 ± 0.03 |

[a]Based on 28–41 tumours. [b]The slope of linear curves fitted to double logarithmic plots of $^{86}$Rb uptake per viable tumour cell (% of injected/cell) *vs* tumour volume (mm$^3$). [c]Mean ± s.e.

mined by linear regression analysis. A significance level of $P = 0.05$ was used throughout.

### Results

### *$^{31}$P-NMR spectra*

Typical $^{31}$P-NMR spectra of small SAX-t and WIX-t tumours are shown in Figure 1. The tumours of all lines showed qualitatively similar spectra. In some spectra, particularly from large tumours, no PCr resonance could be seen. The PME and PDE resonances often appeared as doublets. The resonance assignment in Figure 1 is in accordance with results from analyses of perchloric acid tumour extracts (Evanochko *et al.*, 1984*b*; Corbett *et al.*, 1987).

**Figure 1** $^{31}$P-NMR spectra of a phosphorus-free gel material implanted s.c. in the flank of a mouse **a**, a small tumour of the SAX-t human melanoma xenograft line ($V = 270\ mm^3$) **b**, and a small tumour of the WIX-t human melanoma xenograft line ($V = 180\ mm^3$) **c**. The spectra in **b** and **c** show seven clear, major peaks corresponding to PME, $P_i$, PDE, PCr, NTPγ, NTPα and NTPβ. In **b** the resonance ratios were calculated to be 0.13 (PME/total), 0.18 ($P_i$/total), 0.06 (PDE/total), 0.09 (PCr/total), 0.16 (NTPγ/total), 0.20 (NTPα/total) and 0.17 (NTPβ/total). Tumour pH was determined to be 7.07. In **c** the resonance ratios were calculated to be 0.18 (PME/total), 0.10 ($P_i$/total), 0.07 (PDE/total), 0.08 (PCr/total), 0.20 (NTPγ/total), 0.20 (NTPα/total) and 0.18 (NTPβ/total). Tumour pH was determined to be 7.45.

The PME/total, $P_i$/total, PDE/total, PCr/total, NTPγ/total, NTPα/total, NTPβ/total, (PCr + NTPβ)/total and (PCr + NTPβ)/$P_i$ resonance ratios as well as tumour pH were analysed as a function of tumour volume. The data for one of the xenograft lines are illustrated in Figure 2. Linear curves in semilogarithmic diagrams gave good fits to all data sets. The use of a logarithmic volume axis gave better fits than the use of a linear volume axis. The parameters defining the curves in semilogarithmic diagrams are listed in Table II. The resonance ratios at tumour volumes of 200, 500 and 1,000 mm$^3$ were determined from the curves and corrected for effects of partial saturation. The uncorrected and corrected values at 200 mm$^3$ are listed in Table III. Only minor differences were found between the two values (Table III) and the differences were generally even smaller at 500 and 1,000 mm$^3$ (data not shown). The slopes of uncorrected and corrected volume-dependence curves were not significantly different either (data not shown). The standard errors of the corrected resonance ratios, however, were larger than those of the uncorrected resonance ratios due to the error component associated with the measurement of the $T_1$s (Table III). The results presented henceforth are therefore based on uncorrected data. However, no conclusions were drawn unless they were supported by both uncorrected and corrected data.

### *Differences among tumour lines*

Tumour bioenergetic status; i.e. the (PCr + NTPβ)/$P_i$ resonance ratio, was higher for the WIX-t line than for the BEX-t, COX-t, HUX-t, ROX-t and SAX-t lines ($P < 0.05$), whereas the latter lines showed only minor differences in bioenergetic status (Figure 3). The elevated bioenergetic status of the WIX-t line was due to a low level of inorganic phosphate as well as a high level of high-energy phosphates; the $P_i$/total resonance ratio was reduced at tumour volumes of 200 mm$^3$ and 500 mm$^3$ ($P < 0.05$) (Figure 3a) and the (PCr + NTPβ)/total resonance ratio was enhanced at a tumour volume of 1,000 mm$^3$ ($P < 0.05$) (Figure 3b). Consequently, the (PCr + NTPβ)/$P_i$ resonance ratio was elevated at all tumour volumes studied ($P < 0.05$) (Figure 3c). The NTPγ/total and NTPα/total resonance ratios did not differ significantly among the xenograft lines (Table II).

The BEX-t line showed no change in bioenergetic status with increasing tumour volume (Table II). Thus, no change was found in the $P_i$/total and (PCr + NTPβ)/total resonance ratios either. The other lines showed a significant decrease in bioenergetic status with increasing tumour volume ($P < 0.05$) (Table II). The decrease was a consequence of an increase in the $P_i$/total resonance ratio ($P < 0.05$, except for the ROX-t line) as well as a decrease in the (PCr + NTPβ)/total resonance ratio ($P < 0.05$). The decrease in the (PCr + NTPβ)/total resonance ratio was either mainly due to a decrease in the PCr/total resonance ratio (COX-t and HUX-t) or mainly due to a decrease in the NTPβ/total resonance ratio (ROX-t, SAX-t and WIX-t), depending on the tumour line (Figure 4). The NTPγ/total and NTPα/total resonance ratios showed no significant volume-dependence for any of the xenograft lines (Table II).

The PME/total and PDE/total resonance ratios showed only minor differences among the xenograft lines and no significant changes with increasing tumour volume for any line (Table II).

Tumour pH differed among the xenograft lines at small tumour volumes, whereas only insignificant differences were found at large tumour volumes (Table II). Tumour pH was higher for the COX-t, HUX-t, ROX-t and WIX-t lines than for the SAX-t and BEX-t lines at a tumour volume of 500 mm$^3$ ($P < 0.05$) (Figure 3d). The BEX-t line showed a lower pH at a tumour volume of 200 mm$^3$ than the SAX-t line ($P < 0.05$). Moreover, the BEX-t line showed no change in tumour pH with increasing tumour volume, whereas the other lines showed a significant decrease ($P < 0.05$). The magnitude of this decrease differed among the lines (Table II).

**Figure 2** PME/total **a**, $P_i$/total **b**, PDE/total **c**, PCr/total **d**, NTPγ/total **e**, NTPα/total **f**, NTPβ/total **g**, (PCr + NTPβ)/total **h**, (PCr + NTPβ)/$P_i$ **i**, and tumour pH **j**, *vs* tumour volume for the SAX-t human melanoma xenograft line. ○: individual tumours; □,■: repeated spectrum acquisition of individual tumours; △,▲: repeated processing and analysis of individual spectra. Curves: best fits to the data from individual tumours (○).

Previous work has shown that tumour pH is related to bioenergetic status, but different relationships may exist for different tumour lines (Rofstad *et al.*, 1988*d*). Linear curves were fitted to plots of tumour pH *vs* the (PCr + NTPβ)/$P_i$ resonance ratio. The slopes of the curves differed among the lines, as illustrated for the BEX-t and COX-t lines in Figure 5. A significant decrease in tumour pH with decreasing bioenergetic status was found for the COX-t, HUX-t, SAX-t and WIX-t lines ($P < 0.05$). Tumour pH for the BEX-t and ROX-t lines was found to be independent of bioenergetic status.

*Relationship to blood supply*

The xenograft lines showed considerable differences in tumour blood supply (Table I). Blood supply per viable tumour cell decreased with increasing tumour volume for all lines. The magnitude of the decrease differed among the lines and was smallest for the BEX-t line. The BEX-t line showed no statistically significant change in bioenergetic status with increasing tumour volume. Bioenergetic status decreased with increasing tumour volume for all the other lines (Table II). Thus, there was a relationship between bioenergetic status and blood supply per viable tumour cell within each individual tumour line. No correlation was found between the (PCr + NTPβ)/$P_i$ resonance ratio or any other resonance ratio and blood supply per viable tumour cell across the xenograft lines. However, the magnitude of the decrease in the (PCr + NTPβ)/total resonance ratio was correlated to the magnitude of the decrease in blood supply per viable tumour cell; i.e. the volume-dependence of the (PCr + NTPβ)/total resonance ratio was correlated to the volume-dependence of blood supply per viable tumour cell ($P < 0.05$) (Figure 6a). A similar trend was observed when the (PCr + NTPβ)/$P_i$ resonance ratio was considered, but a statistically significant correlation was not found.

Tumour pH did not show a statistically significant change with increasing tumour volume for the BEX-t line. The other lines, however, showed a decrease in tumour pH with increasing tumour volume (Table II). Thus, a relationship between tumour pH and blood supply per viable tumour cell was found for each individual tumour line. Tumour pH showed no correlation to blood supply per viable tumour cell across the xenograft lines. However, a correlation was found between the magnitude of the decrease in tumour pH and the magnitude of the decrease in blood supply per viable tumour cell; i.e. the volume-dependence of tumour pH was correlated to the volume-dependence of blood supply per viable tumour cell ($P < 0.05$) (Figure 6b).

The correlation between tumour pH and bioenergetic status differed between the different xenograft lines (Figure 5). The slope of the linear curves fitted to plots of tumour pH *vs* bioenergetic status was positively correlated to blood supply per viable tumour cell, independent of whether blood supply per viable tumour cell was measured at tumour volumes of 200, 500 or 1,000 $mm^3$ (Figure 7). Thus, the xenograft lines showing a high blood supply per viable tumour cell (COX-t, HUX-t, SAX-t) showed a large decrease in tumour pH with decreasing bioenergetic status, whereas the lines showing the low blood supply per viable tumour cell (ROX-t, SAX-t, WIX-t) showed only a small or no decrease in tumour pH with decreasing bioenergetic status.

### Discussion

*Methodological aspects*

The $^{31}$P-NMR spectra of the human melanoma xenografts studied here were qualitatively similar to those reported for other human tumour xenografts (Evanochko *et al.*, 1982; Rofstad *et al.*, 1988*b*). The tumours showed significant levels of PCr even at large volumes. It has been suggested that PCr resonances in tumour spectra are caused by adjacent muscle tissue or overlying skin rather than by the tumour tissue itself (Irving *et al.*, 1985). Connective tissue, infiltrating lym-

**Table II** Volume-dependence of $^{31}$P-NMR resonance ratios for human melanoma xenograft lines

| *$^{31}$P-NMR resonance ratio* | *BEX-t* Slope[a] | *BEX-t* Y-intersect[b] | *COX-t* Slope | *COX-t* Y-intersect | *HUX-t* Slope | *HUX-t* Y-intersect | *ROX-t* Slope | *ROX-t* Y-intersect | *SAX-t* Slope | *SAX-t* Y-intersect | *WIX-t* Slope | *WIX-t* Y-intersect |
|---|---|---|---|---|---|---|---|---|---|---|---|---|
| PME/total | − 0.02 ± 0.03[c] | 0.22 ± 0.08 | 0.01 ± 0.02 | 0.13 ± 0.06 | 0.02 ± 0.02 | 0.08 ± 0.05 | 0.04 ± 0.04 | 0.12 ± 0.12 | − 0.01 ± 0.02 | 0.18 ± 0.05 | 0.02 ± 0.02 | 0.10 ± 0.07 |
| $P_i$/total | 0.00 ± 0.03 | 0.18 ± 0.07 | 0.06 ± 0.02 | 0.02 ± 0.05 | 0.09 ± 0.03 | − 0.04 ± 0.07 | 0.05 ± 0.03 | 0.02 ± 0.09 | 0.11 ± 0.03 | − 0.09 ± 0.08 | 0.09 ± 0.02 | − 0.12 ± 0.05 |
| PDE/total | 0.02 ± 0.02 | 0.04 ± 0.06 | 0.00 ± 0.02 | 0.10 ± 0.05 | − 0.02 ± 0.02 | 0.17 ± 0.06 | − 0.03 ± 0.04 | 0.17 ± 0.10 | − 0.01 ± 0.02 | 0.11 ± 0.06 | − 0.02 ± 0.02 | 0.14 ± 0.05 |
| PCr/total | − 0.04 ± 0.02 | 0.19 ± 0.07 | − 0.06 ± 0.02 | 0.23 ± 0.05 | − 0.08 ± 0.02 | 0.28 ± 0.05 | − 0.02 ± 0.02 | 0.14 ± 0.06 | − 0.02 ± 0.02 | 0.14 ± 0.05 | − 0.01 ± 0.02 | 0.13 ± 0.05 |
| NTPγ/total | 0.05 ± 0.02 | 0.06 ± 0.06 | 0.02 ± 0.02 | 0.13 ± 0.05 | 0.01 ± 0.02 | 0.16 ± 0.06 | − 0.02 ± 0.02 | 0.22 ± 0.06 | − 0.02 ± 0.01 | 0.23 ± 0.04 | − 0.02 ± 0.01 | 0.24 ± 0.03 |
| NTPα/total | − 0.01 ± 0.02 | 0.22 ± 0.05 | 0.01 ± 0.01 | 0.15 ± 0.04 | − 0.02 ± 0.02 | 0.24 ± 0.05 | 0.01 ± 0.02 | 0.16 ± 0.06 | − 0.02 ± 0.02 | 0.25 ± 0.04 | 0.03 ± 0.01 | 0.29 ± 0.03 |
| NTPβ/total | 0.00 ± 0.02 | 0.12 ± 0.05 | − 0.01 ± 0.01 | 0.16 ± 0.04 | 0.00 ± 0.02 | 0.15 ± 0.05 | − 0.05 ± 0.02 | 0.25 ± 0.06 | − 0.03 ± 0.02 | 0.23 ± 0.04 | − 0.03 ± 0.01 | 0.24 ± 0.04 |
| (PCr + NTPβ)/total | − 0.04 ± 0.03 | 0.30 ± 0.07 | − 0.07 ± 0.02 | 0.38 ± 0.06 | − 0.08 ± 0.02 | 0.42 ± 0.06 | − 0.08 ± 0.03 | 0.43 ± 0.07 | − 0.06 ± 0.02 | 0.38 ± 0.06 | − 0.04 ± 0.02 | 0.36 ± 0.05 |
| (PCr + NTPβ)/$P_i$ | − 0.25 ± 0.32 | 2.01 ± 0.87 | − 0.72 ± 0.20 | 3.07 ± 0.54 | − 0.92 ± 0.20 | 3.58 ± 0.55 | − 0.99 ± 0.38 | 4.19 ± 1.06 | − 0.87 ± 0.26 | 3.54 ± 0.69 | − 1.61 ± 0.45 | 6.66 ± 1.28 |
| pH | 0.04 ± 0.09 | 6.99 ± 0.23 | − 0.43 ± 0.10 | 8.40 ± 0.26 | − 0.41 ± 0.12 | 8.34 ± 0.32 | − 0.40 ± 0.17 | 8.32 ± 0.47 | − 0.31 ± 0.09 | 7.93 ± 0.24 | − 0.33 ± 0.07 | 8.09 ± 0.20 |

[a]The slope of linear curves fitted to semilogarithmic plots of resonance ratios *vs* tumour volume ($mm^3$). Based on 27–33 tumours in the volume range of 100–2,000 $mm^3$. [b]The intersect with the ordinate; i.e. log(volume) = 0. [c]Mean ± s.e.

**Table III** $^{31}$P-NMR resonance ratios for human melanoma xenograft lines

| *$^{31}$P-NMR resonance ratio* | *BEX-t* $P_{uncorr}$[a] | *BEX-t* $P_{corr}$[b] | *COX-t* $P_{uncorr}$ | *COX-t* $P_{corr}$ | *HUX-t* $P_{uncorr}$ | *HUX-t* $P_{corr}$ | *ROX-t* $P_{uncorr}$ | *ROX-t* $P_{corr}$ | *SAX-t* $P_{uncorr}$ | *SAX-t* $P_{corr}$ | *WIX-t* $P_{uncorr}$ | *WIX-t* $P_{corr}$ |
|---|---|---|---|---|---|---|---|---|---|---|---|---|
| PME/total | 0.18 ± 0.01[c] | 0.18 ± 0.03 | 0.16 ± 0.01 | 0.18 ± 0.02 | 0.14 ± 0.01 | 0.13 ± 0.01 | 0.20 ± 0.02 | 0.18 ± 0.02 | 0.16 ± 0.01 | 0.16 ± 0.02 | 0.15 ± 0.01 | 0.19 ± 0.01 |
| $P_i$/total | 0.19 ± 0.01 | 0.20 ± 0.03 | 0.17 ± 0.01 | 0.18 ± 0.02 | 0.16 ± 0.01 | 0.19 ± 0.02 | 0.13 ± 0.02 | 0.13 ± 0.02 | 0.16 ± 0.01 | 0.16 ± 0.02 | 0.08 ± 0.01 | 0.08 ± 0.01 |
| PDE/total | 0.08 ± 0.01 | 0.09 ± 0.02 | 0.09 ± 0.01 | 0.07 ± 0.01 | 0.11 ± 0.01 | 0.11 ± 0.01 | 0.09 ± 0.02 | 0.10 ± 0.02 | 0.07 ± 0.01 | 0.07 ± 0.01 | 0.10 ± 0.01 | 0.11 ± 0.02 |
| PCr/total | 0.09 ± 0.01 | 0.10 ± 0.01 | 0.09 ± 0.01 | 0.10 ± 0.02 | 0.10 ± 0.01 | 0.09 ± 0.01 | 0.09 ± 0.01 | 0.10 ± 0.01 | 0.09 ± 0.01 | 0.11 ± 0.02 | 0.11 ± 0.01 | 0.13 ± 0.02 |
| NTPγ/total | 0.16 ± 0.01 | 0.15 ± 0.02 | 0.16 ± 0.01 | 0.16 ± 0.02 | 0.17 ± 0.01 | 0.15 ± 0.01 | 0.17 ± 0.01 | 0.16 ± 0.01 | 0.18 ± 0.01 | 0.16 ± 0.02 | 0.19 ± 0.01 | 0.15 ± 0.01 |
| NTPα/total | 0.20 ± 0.01 | 0.18 ± 0.02 | 0.18 ± 0.01 | 0.16 ± 0.02 | 0.20 ± 0.01 | 0.21 ± 0.02 | 0.18 ± 0.01 | 0.19 ± 0.01 | 0.20 ± 0.01 | 0.18 ± 0.02 | 0.22 ± 0.01 | 0.20 ± 0.01 |
| NTPβ/total | 0.13 ± 0.01 | 0.10 ± 0.02 | 0.14 ± 0.01 | 0.15 ± 0.02 | 0.14 ± 0.01 | 0.13 ± 0.01 | 0.15 ± 0.01 | 0.14 ± 0.01 | 0.15 ± 0.01 | 0.15 ± 0.02 | 0.16 ± 0.01 | 0.15 ± 0.01 |
| (PCr + NTPβ)/total | 0.22 ± 0.01 | 0.20 ± 0.02 | 0.23 ± 0.01 | 0.25 ± 0.03 | 0.23 ± 0.01 | 0.22 ± 0.01 | 0.25 ± 0.01 | 0.24 ± 0.01 | 0.25 ± 0.01 | 0.26 ± 0.03 | 0.27 ± 0.01 | 0.28 ± 0.02 |
| (PCr + NTPβ)/$P_i$ | 1.43 ± 0.16 | 1.00 ± 0.18 | 1.43 ± 0.09 | 1.39 ± 0.23 | 1.47 ± 0.10 | 1.16 ± 0.13 | 1.93 ± 0.19 | 1.85 ± 0.29 | 1.55 ± 0.11 | 1.63 ± 0.28 | 2.96 ± 0.27 | 3.50 ± 0.36 |

[a]Resonance ratio at $V = 200\ mm^3$. [b]Resonance ratio at $V = 200\ mm^3$ corrected for effects of partial saturation. [c]Mean ± s.e. s.e. $= s \cdot \sqrt{(1/n) + (x-\bar{x})^2/s_x^2}$, where $s^2 = SSE/(n-2)$, $n$ = number of tumours and $x$ = log(volume). Based on 27–33 tumours in the volume range of 100–2,000 $mm^3$.

**Figure 3** Ranges (mean ± s.e.) for $P_i$/total **a**, (PCr + NTPβ)/total **b**, (PCr + NTPβ)/$P_i$ **c**, and tumour pH **d**, *vs* tumour volume for six human melanoma xenograft lines (BEX-t, COX-t, HUX-t, ROX-t, SAX-t and WIX-t).

**Figure 4** The magnitude of the decrease in PCr/total **a**, and NTPβ/total **b**, with increasing tumour volume for six human melanoma xenograft lines (BEX-t, COX-t, HUX-t, ROX-t, SAX-t and WIX-t). Bars: s.e. In **a** the decrease was statistically significant for the COX-t ($P<0.005$) and HUX-t ($P<0.0005$) lines. In **b** the decrease was statistically significant for the ROX-t ($P<0.05$), SAX-t ($P<0.05$) and WIX-t ($P<0.05$) lines.

phocytes and endothelial cells may also contribute to the PCr resonance. The use of a copper foil Faraday shield and coils fitting closely around the tumours prevented spectrum contamination by signals from adjacent tissues in the present work. Thus, $^{31}$P-NMR spectra of phosphorus-free phantoms showed no mobile phosphates (Figure 1a). Moreover, histological analyses have shown that the melanoma xenografts contain only small amounts of connective tissue, and the infiltration of lymphocytes is sparse (Rofstad *et al.*, 1990). The PCr resonance was therefore probably caused mainly by the melanoma cells themselves.

The $^{31}$P-NMR resonance ratios were corrected for effects of partial saturation (Table III). Only minor differences were found between the uncorrected and corrected values. The WIX-t line showed the largest differences; e.g. the uncorrected value for the PME/total resonance ratio was 0.15 ± 0.01 at a tumour volume of 200 mm$^3$, whereas the corrected value was 0.19 ± 0.01. The differences between the slopes of uncorrected and corrected curves describing the volume-dependence of the resonance ratios were also small. The largest differences were found for the BEX-t line; e.g. the slopes of the uncorrected and corrected curves for the NTPβ/total resonance ratio were 0.01 ± 0.02 and 0.06 ± 0.02, respectively. The differences in the $T_1$s between resonances were thus not large enough to cause major differences between uncorrected and corrected parameters at a repetition time of 2 s.

The $^{31}$P-NMR resonance ratios differed considerably among individual tumours of the same xenograft line even when tumours were of similar size (Figure 2). Repetitive

**Figure 5** Tumour pH *vs* (PCr + NTPβ)/$P_i$ for the BEX-t **a**, and COX-t **b**, human melanoma xenograft lines. Points: individual tumours. Curves: linear regression lines ($r = 0.16$, $P > 0.4$ in **a** and $r = 0.55$, $P < 0.005$ in **b**).

acquisition of spectra from the same tumours and repetitive analyses of the same spectra showed that the experimental uncertainties were small (Figure 2), demonstrating that metabolic differences among individual tumours contributed significantly to the variability observed. This conclusion is in agreement with conclusions from studies of the metabolism of other human tumour xenografts (Kallinowski *et al.*, 1988; 1989).

*Biological aspects*

The PME/total and PDE/total resonance ratios did not differ significantly among the xenograft lines. On the other hand, the xenograft lines showed large differences in tumour volume-doubling time and fraction of cells in S-phase (Lyng *et al.*, 1992). Tissue concentrations of phospholipids are associated with the rate of cell membrane synthesis and degradation (Miceli *et al.*, 1988; Radda *et al.*, 1989; Van der Grond *et al.*, 1991). It has been suggested that the PME and/or PDE resonances of $^{31}$P-NMR spectra of tumours may be utilised to assess the rate of tumour cell proliferation (Smith *et al.*, 1991; Kalra *et al.*, 1993). The data reported here does not support this suggestion. However, differences in volume-doubling time and fraction of cells in S-phase do not necessarily reflect differences in rate of cell proliferation. Moreover, a magnetic field strength of 4.7 T may be sup-optimal for the detection of differences in PME and PDE resonances among tumour lines (Lowry *et al.*, 1992).

The bioenergetic status and the pH of the human melanoma xenografts were within the same ranges as those

**Figure 6** The magnitude of the decrease in (PCr + NTPβ)/total **a**, and tumour pH **b**, with increasing tumour volume *vs* the magnitude of the decrease in blood supply per viable tumour cell with increasing tumour volume for six human melanoma xenograft lines (BEX-t, COX-t, HUX-t, ROX-t, SAX-t and WIX-t). Points: mean values. Bars: s.e. Curves: weighted linear regression lines ($r = 0.79$, $P < 0.05$ in **a** and $r = 0.96$, $P < 0.05$ in **b**).

**Figure 7** The slope of linear curves fitted to plots of tumour pH *vs* tumour bioenergetic status [(PCr + NTPβ)/$P_i$] (Figure 5) *vs* blood supply per viable tumour cell at tumour volumes of 200 mm$^3$ **a**, 500 mm$^3$ **b**, and 1,000 mm$^3$ **c**, for six human melanoma xenograft lines (BEX-t, COX-t, HUX-t, ROX-t, SAX-t and WIX-t). Points: mean values. Bars: s.e. Curves: weighted linear regression lines ($r = 0.94$, $P < 0.005$ in **a** and **b**, $r = 0.92$, $P < 0.01$ in **c**).

reported for other experimental human tumours (Rofstad et al., 1988b; Vaupel et al., 1989a). The BEX-t line showed no change in bioenergetic status or pH with increasing tumour volume. The other lines showed a decrease in bioenergetic status that was accompanied by a decrease in pH (Table II). Comparable changes in the $^{31}$P-NMR spectrum during tumour growth have been reported for other transplantable tumour lines as well (Evanochko et al., 1982; Okunieff et al., 1986; Rofstad et al., 1988b; Koutcher et al., 1990). The changes have been attributed to changes in the steady state tumour cell metabolism occurring when the cellular oxygen concentration decreases. This is consistent with the observation that blood supply per viable tumour cell decreased with increasing tumour volume for the melanoma xenografts (Table I). Alternatively, it has been suggested that the changes observed in $^{31}$P-NMR energy parameters during tumour growth are a consequence of increasing occurrence of acute hypoxia rather than increasing chronic nutrient deprivation (Freyer et al., 1991).

The (PCr + NTPβ)/$P_i$ resonance ratio was not correlated to blood supply per viable tumour cells across the melanoma xenograft lines. The lack of correlation was probably a consequence of differences in vascular architecture as well as in cellular biochemistry among the lines. Nutritive blood flow is not necessarily correlated to total blood flow across tumour lines, due to differences in capillary branching pattern and occurrence of arteriovenous anastomoses. There is evidence that the vascular architecture differed among the melanoma xenograft lines studied here (Lyng et al., 1992). Nevertheless, nutritive blood flow was probably correlated to blood supply per viable tumour cell; a statistically significant correlation between blood supply per viable tumour cell and fraction of cells in S-phase has been demonstrated (Lyng et al., 1992). The differences in bioenergetic status among the lines were therefore not attributable to differences in nutritive blood flow alone. The WIX-t line showed significantly higher bioenergetic status than the other lines in spite of a low blood supply per viable tumour cell (Figure 3c), possibly because the WIX-t tumours contained a low fraction of metabolically active hypoxic cells. This hypothesis is supported by results from *in vitro* studies performed in our laboratory: (a) the WIX-t cells have been found to show large numbers of mitochondria, high rates of oxygen consumption and poor ability to survive under hypoxic conditions compared with the cells of the other lines; and (b) the thickness of the viable rim of the WIX-t multicellular spheroids has been measured to be shorter than the oxygen diffusion distance. The differences in $^{31}$P-NMR bioenergetic status observed among the melanoma xenograft lines were thus possibly caused by differences in intrinsic biochemical properties of the tumour cells, such as energy demand and tolerance to hypoxic stress, rather than by the differences in blood supply per viable tumour cell.

The (PCr + NTPβ)/total resonance ratio and blood supply per viable tumour cell decreased with increasing tumour volume. The magnitude of the decrease in high-energy phosphates was correlated to the magnitude of the decrease in blood supply per viable tumour cell (Figure 6a). The differences in the magnitude of the decrease in high energy phosphates observed among the melanoma xenograft lines were therefore probably caused mainly by the differences in the magnitude of the decrease in tumour blood supply per viable tumour cell. This conclusion is based on the assumption that the intrinsic biochemical properties of the tumour cells were maintained within the volume range studied.

The decrease in the (PCr + NTPβ)/total resonance ratio with increasing tumour volume was mainly due to a decrease in the PCr resonance for the COX-t and HUX-t lines and a decrease in the NTPβ resonance for the ROX-t, SAX-t and WIX-t lines (Figure 4). This result can probably be attributed to physiological heterogeneity within the tumours. Metabolic compartments with different levels of high-energy phosphates may exist in tumours due to spatial heterogeneity in blood supply and oxygenation (Sutherland et al., 1988). Tumour cells in well perfused compartments can have high levels of PCr as well as ATP. When the supply of oxygen and glucose is reduced gradually in such compartments, more and more cells will utilise their PCr, thus maintaining the level of ATP while adjusting to new equilibrium states (Tozer & Griffiths, 1992). On the other hand, most cells in poorly perfused compartments are depleted of PCr. The level of ATP will therefore decrease in such compartments when the supply of oxygen and glucose gradually decreases. The present results are consistent with the assumption that the decrease in blood supply with increasing tumour volume occurred mainly in the first type of compartment for the COX-t and HUX-t lines and mainly in the second type of compartment for the ROX-t, SAX-t and WIX-t lines.

Tumour pH differed among the xenograft lines, but the differences could not be attributed to differences in tumour blood supply alone; no correlation was found between tumour pH and blood supply per viable tumour cell across the lines. The BEX-t and SAX-t lines showed lower pH at small tumour volumes than the other lines. The low pH of the BEX-t line was probably a consequence of a low blood supply per viable tumour cell (Table I). The SAX-t line, however, also showed low pH in spite of a high blood supply per viable tumour cell (Table I). Studies *in vitro* have shown that the SAX-t cells have high glycolytic capacity compared with cells of the other lines. Thus, differences in intrinsic biochemical properties of the tumour cells may have contributed significantly to the differences in tumour pH observed among the lines.

Tumour pH and blood supply per viable tumour cell decreased with increasing tumour volume. The magnitude of the decrease in tumour pH was correlated to the magnitude of the decrease in blood supply per viable tumour cell (Figure 6b). The differences in the magnitude of the decrease in tumour pH observed among the melanoma xenograft lines were therefore probably caused mainly by the differences in the magnitude of the decrease in blood supply per viable tumour cell. This conclusion is based on the assumption that the glycolytic capacity of the tumour cells showed only minor changes within the volume range studied.

The magnitude of the decrease in tumour pH with decreasing bioenergetic status was larger for the xenograft lines showing high than for those showing low blood supply per viable tumour cell (Figure 7). It is possible that a relatively large decrease in the blood supply is needed to cause a decrease in bioenergetic status for the lines showing high blood supply per viable tumour cell. Tumour pH may thus decrease considerably because the transport of $H^+$ ions out of the tumour is reduced. In contrast, a minor decrease in the blood supply may cause a decrease in bioenergetic status for the lines showing low blood supply per viable tumour cell. Consequently, only minor changes in tumour pH may occur. The $^{86}$Rb uptake and $^{31}$P-NMR spectroscopy data (Tables I–III) confirm the validity of the relationships between blood supply per viable tumour cell and bioenergetic status assumed here.

Tumour response to radiation therapy, hyperthermia, chemotherapy and photodynamic therapy depends partly on physiological conditions in the tumour that are mainly determined by the tumour blood supply. Tumour $^{31}$P-NMR bioenergetic status was found to be correlated to blood supply per viable tumour cell within individual melanoma xenograft lines. This observation is consistent with previous results showing a relationship between $^{31}$P-NMR resonance ratios and tumour oxygenation. Thus, Evelhoch et al. (1986) found that the PCr/NTPβ and NTPβ/$P_i$ resonance ratios were correlated to the $^{15}$O perfusion in the well perfused tumour fraction using a mouse fibrosarcoma line. Similar relationships have been reported between $^{31}$P-NMR resonance ratios related to tumour energy status and oxygen tension (Okunieff et al., 1987; Vaupel et al., 1989b; Sostman et al., 1991), radiobiologic hypoxic fraction (Rofstad et al., 1988a; Wendland et al., 1992), and oxyhemoglobin ($HbO_2$) saturation status (Rofstad et al., 1988a,c). The present results give further support to the suggestion that $^{31}$P-NMR spectroscopy may be a clinically useful method for monitoring tumour blood supply and parameters related to blood supply during and after physiological intervention and tumour treatment.

It has been suggested that $^{31}$P-NMR resonance ratios may be used to predict tumour treatment response as well (Ng *et al.*, 1982; Evanochko *et al.*, 1983; 1984*a*). However, prediction of treatment response requires correlations between $^{31}$P-NMR resonance ratios and physiological parameters across tumour lines. Rofstad *et al.* (1988*a*) found no correlations between $^{31}$P-NMR resonance ratios and radiobiologic hypoxic fraction or $HbO_2$ saturation status across tumour lines. However, two human ovarian carcinoma and two murine fibrosarcoma lines were used in their study; i.e. the tumour lines were of completely different origin. A more homogeneous tumour panel consisting of six lines of the same histological type was used in the present study. No correlations were found between $^{31}$P-NMR resonance ratios and tumour blood supply across these lines either, indicating that clinically useful prediction criteria based on $^{31}$P-NMR resonance ratios may be difficult to find. Consequently, $^{31}$P-NMR resonance ratios probably have to be supplemented with other data to be useful in prediction of tumour treatment response.

The skilful technical assistance of Heidi Kongshaug, Berit Mathiesen and Hanne Stageboe Petersen is gratefully acknowledged. Financial support from The Norwegian Cancer Society is highly appreciated.

### References

BREMNER, J.C.M., COUNSELL, C.J.R., ADAMS, G.E., STRATFORD, I.J., WOOD, P.J., DUNN, J.F. & RADDA, G.K. (1991). *In vivo* $^{31}$P nuclear magnetic resonance spectroscopy of experimental murine tumours and human tumour xenografts: effects of blood flow modification. *Br. J. Cancer*, **64**, 862–866.

CECKLER, T.L., BRYANT, R.G., PENNEY, D.P., GIBSON, S.L. & HILF, R. (1986). $^{31}$P-NMR spectroscopy demonstrates decreased ATP levels *in vivo* as an early response to photodynamic therapy. *Biochem. Biophys. Res. Commun.*, **140**, 273–279.

CHAPMAN, J.D., MCPHEE, M.S., WATZ, N., CHETNER, M.P., STOBBE, C.C., SODERLIND, K., ARNFIELD, M., MEELUR, B.E., TRIMBLE, L. & ALLEN, P.S. (1991). Nuclear magnetic resonance spectroscopy and sensitizer-adduct measurements of photodynamic therapy-induced ischemia in solid tumors. *J. Natl Cancer Inst.*, **83**, 1650–1659.

CORBETT, R.J.T., NUNNALLY, R.L., GIOVANELLA, B.C. & ANTICH, P.P. (1987). Characterization of the $^{31}$P nuclear magnetic resonance spectrum from human melanoma tumors implanted in nude mice. *Cancer Res.*, **47**, 5065–5069.

DALY, P.F. & COHEN, J.S. (1989). Magnetic resonance spectroscopy of tumors and potential *in vivo* applications: a review. *Cancer Res.*, **49**, 770–779.

EVANOCHKO, W.T., NG, T.C. & GLICKSON, J.D. (1984*a*). Applications of *in vivo* NMR spectroscopy to cancer. *Magn. Reson. Med.*, **1**, 508–534.

EVANOCHKO, W.T., NG, T.C., GLICKSON, J.D., DURANT, J.R. & CORBETT, T.H. (1982). Human tumors as examined by *in vivo* $^{31}$P NMR in athymic mice. *Biochem. Biophys. Res. Commun.*, **109**, 1346–1352.

EVANOCHKO, W.T., NG, T.C., LILLY, M.B., LAWSON, A.J., CORBETT, T.H., DURANT, J.R. & GLICKSON, J.D. (1983). *In vivo* $^{31}$P NMR study of the metabolism of murine mammary 16/C adenocarcinoma and its response to chemotherapy, x-radiation, and hyperthermia. *Proc. Natl Acad. Sci. USA*, **80**, 334–338.

EVANOCHKO, W.T., SAKAI, T.T., NG, T.C., KRISHNA, N.R., KIM, H.D., ZEIDLER, R.B., GHANTA, V.K., BROCKMAN, R.W., SCHIFFER, L.M., BRAUNSCHWEIGER, P.G. & GLICKSON, J.D. (1984*b*). NMR study of *in vivo* RIF-1 tumors. Analysis of perchloric acid extracts and identification of $^{1}$H, $^{31}$P and $^{13}$C resonances. *Biochem. Biophys. Acta*, **805**, 104–116.

EVELHOCH, J.L., SAPARETO, S.A., NUSSBAUM, G.H. & ACKERMAN, J.J.H. (1986). Correlations between $^{31}$P NMR spectroscopy and $^{15}$O perfusion measurements in the RIF-1 murine tumor *in vivo*. *Radiat. Res.*, **106**, 122–131.

FREYER, J.P., SCHOR, P.L., JARRETT, K.A., NEEMAN, M. & SILLERUD, L.O. (1991). Cellular energetics measured by phosphorus nuclear magnetic resonance spectroscopy are not correlated with chronic nutrient deficiency in multicellular tumor spheroids. *Cancer Res.*, **51**, 3831–3837.

GODFREY, K. (1985). Statistics in practice. Comparing the means of several groups. *N. Engl. J. Med.*, **313**, 1450–1456.

IRVING, M.G., SIMPSON, S.J., FIELD, J. & DODDRELL, D.M. (1985). Use of high-resolution $^{31}$P-labeled topical magnetic resonance spectroscopy to monitor *in vivo* tumor metabolism in rats. *Cancer Res.*, **45**, 481–486.

KALLINOWSKI, F., SCHLENGER, K.H., RUNKEL, S., KLOES, M., STOHRER, M., OKUNIEFF, P. & VAUPEL, P. (1989). Blood flow, metabolism, cellular microenvironment, and growth rate of human tumor xenografts. *Cancer Res.*, **49**, 3759–3764.

KALLINOWSKI, F., VAUPEL, P., RUNKEL, S., BERG, G., FORTMEYER, H.P., BAESSLER, K.H., WAGNER, K., MUELLER-KLIESER, W. & WALENTA, S. (1988). Glucose uptake, lactate release, ketone body turnover, metabolic micromilieu, and pH distributions in human breast cancer xenografts in nude rats. *Cancer Res.*, **48**, 7264–7272.

KALRA, R., WADE, K.E., HANDS, L., STYLES, P., CAMPLEJOHN, R., GREENALL, M., ADAMS, G.E., HARRIS, A.L. & RADDA, G.K. (1993). Phosphomonoester is associated with proliferation in human breast cancer: a $^{31}$P MRS study. *Br. J. Cancer*, **67**, 1145–1153.

KLUGE, M., ELGER, B., ENGEL, T., SCHAEFER, C., SEEGA, J. & VAUPEL, P. (1992). Acute effects of tumour necrosis factor α or lymphotoxin on global blood flow, laser doppler flux, and bioenergetic status of subcutaneous rodent tumors. *Cancer Res.*, **52**, 2167–2173.

KOUTCHER, J.A., ALFIERI, A.A., BARNETT, D.C., COWBURN, D.C., KORNBLITH, A.B. & KIM, J.H. (1990). Changes in $^{31}$P nuclear magnetic resonance with tumor growth in radioresistant and radiosensitive tumours. *Radiat. Res.*, **121**, 312–319.

KOUTCHER, J.A., ALFIERI, A.A., DEVITT, M.L., RHEE, J.G., KORNBLITH, A.B., MAHMOOD, U., MERCHANT, T.E. & COWBURN, D. (1992). Quantitative changes in tumor metabolism, partial pressure of oxygen, and radiobiological oxygenation status postradiation. *Cancer Res.*, **52**, 4620–4627.

LILLY, M.B., NG, T.C., EVANOCHKO, W.T., KATHOLI, C.R., KUMAR, N.G., ELGAVISH, G.A., DURANT, J.R., HIRAMOTO, R., GHANTA, V. & GLICKSON, J.D. (1984). Loss of high-energy phosphate following hyperthermia demonstrated by *in vivo* $^{31}$P-nuclear magnetic resonance spectroscopy. *Cancer Res.*, **44**, 633–638.

LOWRY, M., PORTER, D.A., TWELVES, C.J., HEASLEY, P.E., SMITH, M.A. & RICHARDS, M.A. (1992). Visibility of phospholipids in $^{31}$P NMR spectra of human breast tumours *in vivo*. *NMR Biomed.*, **5**, 37–42.

LYNG, H., SKRETTING, A. & ROFSTAD, E.K. (1992). Blood flow in six human melanoma xenograft lines with different growth characteristics. *Cancer Res.*, **52**, 584–592.

MICELI, M.V., KAN, L. & NEWSOME, D.A. (1988). Phosphorus-31 nuclear magnetic resonance spectroscopy of human retinoblastoma cells: correlations with metabolic indices. *Biochim. Biophys. Acta*, **970**, 262–269.

NARUSE, S., HIRAKAWA, K., HORIKAWA, Y., TANAKA, C., HIGUCHI, T., UEDA, S., NISHIKAWA, H. & WATARI, H. (1985). Measurements of *in vivo* $^{31}$P nuclear magnetic resonance spectra in neuroectodermal tumors for the evaluation of the effects of chemotherapy. *Cancer Res.*, **45**, 2429–2433.

NG, T.C., EVANOCHKO, W.T., HIRAMOTO, R.N., GHANTA, V.K., LILLY, M.B., LAWSON, A.J., CORBETT, T.H., DURANT, J.R. & GLICKSON, J.D. (1982). $^{31}$P NMR spectroscopy of *in vivo* tumors. *J. Magn. Reson.*, **49**, 271–286.

OKUNIEFF, P., KALLINOWSKI, F., VAUPEL, P. & NEURINGER, L.J. (1988). Effects of hydralazine-induced vasodilation on the energy metabolism of murine tumors studied by *in vivo* $^{31}$P-nuclear magnetic resonance spectroscopy. *J. Natl Cancer Inst.*, **10**, 745–750.

OKUNIEFF, P.G., KOUTCHER, J.A., GERWECK, L., MCFARLAND, E., HITZIG, B., URANO, M., BRADY, T., NEURINGER, L. & SUIT, H.D. (1986). Tumor size dependent changes in a murine fibrosarcoma: use of *in vivo* $^{31}$P NMR for non-invasive evaluation of tumour metabolic status. *Int. J. Radiat. Oncol. Biol. Phys.*, **12**, 793–799.

OKUNIEFF, P., MCFARLAND, E., RUMMENY, E., WILLETT, C., HITZIG, B., NEURINGER, L. & SUIT, H. (1987). Effects of oxygen on the metabolism of murine tumors using *in vivo* phosphorus-31 NMR. *Am. J. Clin. Oncol. (CCT)*, **10**, 475–482.

OKUNIEFF, P., VAUPEL, P., SEDLACEK, R. & NEURINGER, L.J. (1989). Evaluation of tumor energy metabolism and microvascular blood flow after glucose or mannitol administration using $^{31}$P nuclear magnetic resonance spectroscopy and laser doppler flowmetry. *Int. J. Radiat. Oncol. Biol. Phys.*, **16**, 1493–1500.

OLSEN, D.R., LYNG, H., SOUTHON, T.E. & ROFSTAD, E.K. (1993). $^{31}$P-nuclear magnetic resonance spectroscopy *in vivo* of six human melanoma xenograft lines: spin-lattice relaxation times. (submitted).

RADDA, G.K., RAJAGOPALAN, B. & TAYLOR, D.J. (1989). Biochemistry *in vivo*: an appraisal of clinical magnetic resonance spectroscopy. *Magn. Reson. Q.*, **5**, 122-151.

ROFSTAD, E.K. (1990). NMR spectroscopy in prediction and monitoring of radiation response of tumours *in vivo*. *Int. J. Radiat. Biol.*, **57**, 1-5.

ROFSTAD, E.K., DEMUTH, P., FENTON, B.M. & SUTHERLAND, R.M. (1988*a*). $^{31}$P nuclear magnetic resonance spectroscopy studies of tumor energy metabolism and its relationship to intracapillary oxyhemoglobin saturation status and tumour hypoxia. *Cancer Res.*, **48**, 5440-5446.

ROFSTAD, E.K., DEMUTH, P. & SUTHERLAND, R.M. (1988*b*). $^{31}$P NMR spectroscopy measurements of human ovarian carcinoma xenografts: relationship to tumour volume, growth rate, necrotic fraction and differentiation status. *Radiother. Oncol.*, **12**, 315-326.

ROFSTAD, E.K., FENTON, B.M. & SUTHERLAND, R.M. (1988*c*). Intracapillary $HbO_2$ saturations in murine tumours and human tumour xenografts measured by cryospectrophotometry: relationship to tumour volume, tumour pH and fraction of radiobiologically hypoxic cells. *Br. J. Cancer*, **57**, 494-502.

ROFSTAD, E.K., HOWELL, R.L., DEMUTH, P., CECKLER, T.L. & SUTHERLAND, R.M. (1988*d*). $^{31}$P NMR spectroscopy *in vivo* of two murine tumor lines with widely different fractions of radiobiologically hypoxic cells. *Int. J. Radiat. Biol.*, **54**, 635-649.

ROFSTAD, E.K., WAHL, A., STOKKE, T. & NESLAND, J.M. (1990). Establishment and characterization of six human melanoma xenograft lines. *Acta Pathol. Microbiol. Immunol. Scand.*, **98**, 945-953.

SIJENS, P.E., BOVÉE, W.M.M.J., KOOLE, P. & SCHIPPER, J. (1989). Phosphorus NMR study of the response of a murine tumour to hyperthermia as a function of treatment time and temperature. *Int. J. Hypertherm.*, **5**, 351-357.

SIJENS, P.E., BOVÉE, W.M.M.J., SEIJKENS, D., LOS, G. & RUTGERS, D.H. (1986). *In vivo* $^{31}$P-nuclear magnetic resonance study of the response of a murine mammary tumor to different doses of γ-radiation. *Cancer Res.*, **46**, 1427-1432.

SMITH, T.A.D., ECCLES, S., ORMEROD, M.G., TOMBS, A.J., TITLEY, J.C. & LEACH, M.O. (1991). The phosphocholine and glycerophosphocholine content of an oestrogen-sensitive ràt mammary tumour correlates strongly with growth rate. *Br. J. Cancer*, **64**, 821-826.

SOSTMAN, H.D., ROCKWELL, S., SYLVIA, A.L., MADWED, D., COFER, G., CHARLES, H.C., NEGRO-VILAR, R. & MOORE, D. (1991). Evaluation of BA1112 Rhabdomyosarcoma oxygenation with microelectrodes, optical spectrophotometry, radiosensitivity, and magnetic resonance spectroscopy. *Magn. Reson. Med.*, **20**, 253-267.

STEEN, R.G. (1989). Response of solid tumors to chemotherapy monitored by *in vivo* $^{31}$P-nucelar magnetic resonance spectroscopy: a review. *Cancer Res.*, **49**, 4075-4085.

STEEN, R.G. (1991). Characterization of tumor hypoxia by $^{31}$P MR spectroscopy. *Am. J. Roentgenol.*, **157**, 243-248.

SUTHERLAND, R.M., RASEY, J.S. & HILL, R.P. (1988). Tumor biology. *Am. J. Clin. Oncol.*, **11**, 253-274.

TANNOCK, I.F. & ROTIN, D. (1989). Acid pH in tumors and its potential for therapeutic exploitation. *Cancer Res.*, **49**, 4373-4384.

TOZER, G.M., BHUJWALLA, Z.M., GRIFFITHS, J.R. & MAXWELL, R.J. (1989). Phosphorus-31 magnetic resonance spectroscopy and blood perfusion of the RIF-1 tumor following X-irradiation. *Int. J. Radiat. Oncol. Biol. Phys.*, **16**, 155-164.

TOZER, G.M. & GRIFFITHS, J.R. (1992). The contribution made by cell death and oxygenation to $^{31}$P MRS observations of tumour energy metabolism. *NMR Biomed.*, **5**, 279-289.

TOZER, G.M., MAXWELL, R.J., GRIFFITHS, J.R. & PHAM, P. (1990). Modification of the $^{31}$P magnetic resonance spectra of a rat tumour using vasodilators and its relationship to hypotension. *Br. J. Cancer*, **62**, 553-560.

VAN DER GROND, J., DIJKSTRA, G., ROELOFSEN, B. & MALI, P.T.M. (1991). $^{31}$P-NMR determination of phosphomonoesters in relation to phospholipid biosynthesis in testis of the rat at different ages. *Biochim. Biophys. Acta*, **1074**, 189-194.

VAUPEL, P., KALLINOWSKI, F. & OKUNIEFF, P. (1989*a*). Blood flow, oxygen and nutrient supply, and metabolic microenvironment of human tumors: a review. *Cancer Res.*, **49**, 6449-6465.

VAUPEL, P., OKUNIEFF, P., KALLINOWSKI, F. & NEURINGER, L.J. (1989*b*). Correlations between $^{31}$P-NMR spectroscopy and tissue $O_2$ tension measurements in a murine fibrosarcoma. *Radiat. Res.*, **120**, 477-493.

VAUPEL, P., OKUNIEFF, P. & NEURINGER, L.J. (1990). *In vivo* $^{31}$P-NMR spectroscopy of murine tumours before and after localized hyperthermia. *Int. J. Hypertherm.*, **6**, 15-31.

WEDLAND, M.F., IYER, S.B., FU, K.K., LAM, K.N. & JAMES, T.L. (1992). Correlations between *in vivo* $^{31}$P MRS measurements, tumor size, cell survival, and hypoxic fraction in the murine EMT6 tumor. *Magn. Reson. Med.*, **25**, 217-232.